# Current perspectives on bone metastases in castrate-resistant prostate cancer

**DOI:** 10.1007/s10555-017-9719-4

**Published:** 2018-01-29

**Authors:** Christopher Logothetis, Michael J. Morris, Robert Den, Robert E. Coleman

**Affiliations:** 1 University of Texas–MD Anderson Cancer Center, Houston, TX USA; 2 Memorial Sloan Kettering Cancer Center, Weill Cornell Medicine, New York, NY USA; 3 Sidney Kimmel Cancer Center, Thomas Jefferson University, Philadelphia, PA USA; 4 Weston Park Hospital, University of Sheffield, Sheffield, UK

**Keywords:** Castrate-resistant prostate cancer, Radium-223, Bone metastases, Tumor microenvironment

## Abstract

Prostate cancer is the most frequent noncutaneous cancer occurring in men. On average, men with localized prostate cancer have a high 10-year survival rate, and many can be cured. However, men with metastatic castrate-resistant prostate cancer have incurable disease with poor survival despite intensive therapy. This unmet need has led to recent advances in therapy aimed at treating bone metastases resulting from prostate cancer. The bone microenvironment lends itself to metastases in castrate-resistant prostate cancer, as a result of complex interactions between the microenvironment and tumor cells. The development of ^223^radium dichloride (Ra-223) to treat symptomatic bone metastases has improved survival in men with metastatic castrate-resistant prostate cancer. Moreover, Ra-223 may have effects on the tumor microenvironment that enhance its activity. Ra-223 treatment has been shown to prolong survival, and its effects on the immune system are under investigation. Because prostate cancer affects a sizable portion of the adult male population, understanding how it metastasizes to bone is an important step in advancing therapy. Clinical trials that are underway should yield new information on whether Ra-223 synergizes effectively with immunotherapy agents and whether Ra-223 has enhancing effects on the immune system in patients with prostate cancer.

## Introduction

Prostate cancer is the most frequent noncutaneous cancer in men and is a major health problem in the developed world. It can be anticipated that with improving life span in developing countries, prostate cancer will emerge as a major health problem worldwide. There is a wide range of outcomes based on the extent of cancer at diagnosis. Overall, men with localized cancer have a high 10-year survival rate, and many can be cured. However, men with metastatic prostate cancer have incurable disease with a poor survival, despite intensive therapy: the 5-year survival rate is only approximately 30%, and median survival rate is approximately 3 years [1, 2].

Resistance to treatment is one factor accounting for the poor survival rate. Several mechanisms have been linked to the emergence of resistance to treatment, including aggressive variants of prostate cancer, which arise as a result of mutations in several tumor suppressor genes [3–6]. In addition, androgen depletion induces genes involved in epithelial-to-mesenchymal transition, which plays a role in cancer progression and metastasis [7]. These observations have implicated the bone–epithelial interaction as a key process in prostate cancer progression.

The bone microenvironment is the natural subject of research interest, given that bone metastases dominate the clinical picture of advanced prostate cancer and form a major source of morbidity from the disease [8]. Crosstalk between tumor cells and osteoblasts, which drives the growth of metastases, is facilitated by soluble factors and by physical contact between the cell types [9, 10]. Investigators hypothesized that the bidirectional interaction between the epithelium and host stromal cells in prostate cancer may account for the development of resistance and unique patterns of spread. Recent experimental results showed that a subset of cancers not expressing the combined loss profile associated with the *p53*, *PTEN*, or *Rb* gene was associated with specific gene expression profiles, including antiapoptotic genes and those promoting tumor spread [11, 12]. Of note, among the genes implicated in prostate carcinogenesis and resistance to therapy, such as matrix metalloproteinase (MMP)-11, androgen receptor, and interleukin (IL)-17 receptor beta, were genes coexpressed by tumor-associated fibroblasts and tumor cells [11].

In contrast to most approved therapies for prostate cancer, the life-prolonging, bone-homing radiopharmaceutical ^223^radium dichloride (Ra-223) and the therapeutic vaccine sipuleucel-T exert their therapeutic benefits with only modest declines in serum prostate-specific antigen (PSA) concentrations [13–15]. Such observations led investigators to speculate that the life-prolonging effects from these agents in the absence of proportional declines in PSA were indirect, the initial target being both immune and nonimmune tumor-associated microenvironments. Taken together, these observations have generated interest in studies linking the mechanism of the development of bone metastases to the benefits of treatment with Ra-223.

Recent data show that Ra-223 affects not only tumor cells but also the bone microenvironment, thereby amplifying the benefits of treatment with this agent. The goal of this review is to discuss the current preclinical and clinical literature, including experimental systems, prevailing hypotheses, and knowledge gaps that should be applied to the novel pathway-driven approaches to the treatment of bone metastases in prostate cancer.

## The bone microenvironment, tumor progression, and metastases in castrate-resistant prostate cancer

Prostate cancer cells home preferentially to osteoblast-rich regions of the bone [16]. The physical contact between prostate cancer cells and osteoblasts in bone disrupts bone structure and develops a cycle of mutually enhanced growth by prostate cancer cells and osteoblasts. In a series of coculture experiments, Kimura and colleagues demonstrated that osteoblasts that were cultured with MDA-PCa-2b cells had increased numbers of both cell types and increased the expression of alkaline phosphatase [9]. Moreover, in the presence of prostate cancer cells, osteoblasts did not align along the collagen matrix in a normal fashion, but rather showed a disorganized arrangement that is not reproduced when cells are cocultured with spent medium from prostate cancer cultures, indicating a need for direct cell contact [9]. This resulting bone matrix anisotropy may enhance prostate cancer metastasis [9].

Hypoxia is a major driver of metastases, and the hypoxic environment of bone induces the expression of hypoxia-inducible factor (HIF)-1. HIF-1 regulates the expression of glycolytic enzymes, glucose transporters, and vascular endothelial growth factor (VEGF) [10]. *In vitro* studies showed that HIF-1α enhanced the invasive potential of human prostate cancer cells and increased the expression of MMP-2 and cathepsin D, both of which are involved in cell migration and invasion [17]. Moreover, hypoxic tissues including neoplasms are generally less susceptible to energetic X-irradiation. However, this should not affect the efficacy of Ra-223, because hypoxia does not modify linear energy transfer in the range of alpha particles [18].

**Fig. 1:**
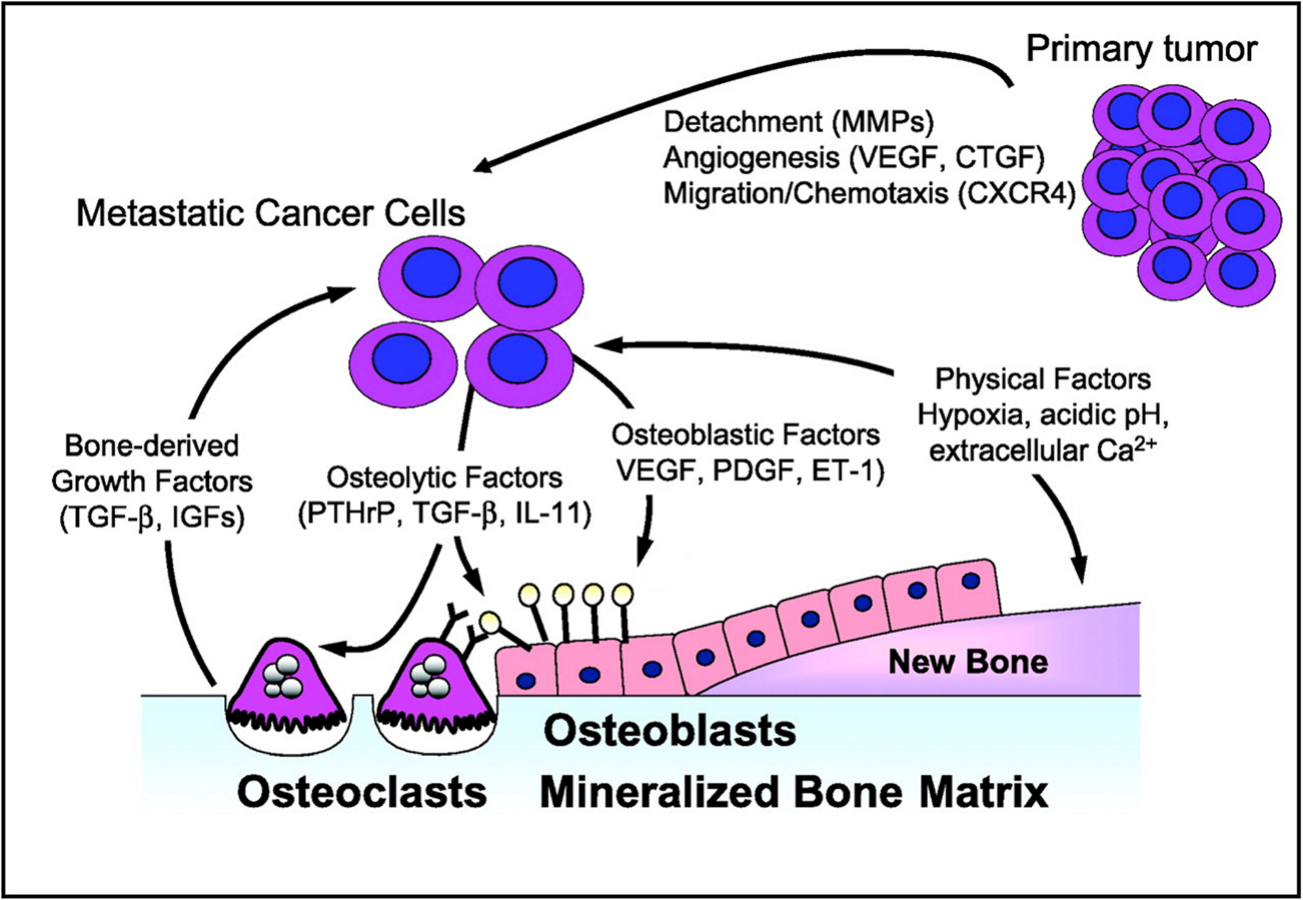
Matrix metalloproteinases (MMPs), chemokine receptor (CXCR)-4, vascular endothelial growth factor (VEGF), and connective tissue growth factor (CTGF) target metastatic tumor cells to bone and facilitate their survival within the bone microenvironment. Physical factors within bone, including hypoxia, acidic pH, and extracellular Ca^2+^, and bone-derived growth factors, such as tumor growth factor (TGF)-β and insulin-like growth factors (IGFs), activate tumor expression of osteoblast-stimulatory factors, such as VEGF, platelet-derived growth factor (PDGF), and endothelin-1 (ET-1). Osteoclast-stimulatory factors can also be increased, which in turn release factors that promote tumor growth in bone. Republished with permission of the American Association of Cancer Research, from [10]. Permission conveyed through Copyright Clearance Center, Inc.

It is generally accepted that bone metastases in prostate cancer are an archetypical example of a specific “seed and soil” hypothesis arising from interactions between tumor cells and the bone microenvironment [19]. This two-compartment model assumes that interactions occur between the tumor and the stromal cell compartment (osteoclasts, osteoblasts T cells, endothelial cells), within bone [20]. Unlike those of many other solid tumors, the bone metastases of metastatic castrate-resistant prostate cancer (CRPC) appear phenotypically osteoblastic rather than osteolytic. Both autocrine and paracrine factors among the various cell types are involved, setting up a vicious cycle that drives metastatic growth (Fig. 1) [21].

Osteoclast stimulatory factors, such as parathyroid hormone-related protein, transforming growth factor (TGF)-β, and IL-11, may also be released, stimulating bone cells and inducing additional factors that promote prostate cancer growth [10, 22]. On balance, bone metastases favor bone erosion more than bone formation when resistance to antiandrogen therapy arises in poorly differentiated or neuroendocrine-type tumors. In a series of experiments, Ottewell and colleagues showed that castrated mice had enhanced bone resorption and subsequent loss of bone volume compared with sham-operated mice. Moreover, castration triggered growth of prostate cancer cells to form bone metastases in 70% of mice, whereas few sham-operated mice had observable bone metastases. By 2 weeks following castration, osteoclast numbers were increased in castrated mice but not in sham-operated mice [8].

Once established, bone metastases enter an autocatalytic vicious cycle of enhancing growth and progression of prostate cancer even as bone metastases develop and grow (Fig. 1). Tumor-derived factors, such as MMPs, chemokine receptor-4, vascular endothelial growth factor (VEGF), and connective tissue growth factor, target metastatic prostate cancer cells to bone and facilitate their survival within it. Physical factors within bone, such as hypoxia, low pH, and extracellular Ca^2^+, activate signaling pathways within prostate cancer cells, leading to the additional release of factors (e.g., TGF-β and insulin-derived growth factors), which, in animal models, enhance survival and growth within bone [10]. Moreover, these factors activate the expression of osteoblast stimulatory factors by prostate cancer cells, including VEGF, platelet-derived growth factor, bone morphogenic protein-2, insulin-like growth factor-1, and endothelin-1 [23–25].

## Patient-derived xenografts and other models to study metastasis formation and guide treatment

Biological models helped elucidate mechanisms underlying cancer progression and resistance to therapy and contributed significantly to modern treatment of many adult solid tumors. Successful approaches have largely focused on genetically engineered mouse models and cell lines suitable for *in vivo* studies. However, the usefulness of these models is restricted to cancers whose growth is driven primarily by cancer cells, without significant contribution from the tumor-associated microenvironment (TME), and furthermore, by the lack of similarity to lesions seen in the clinic: osteoclastic rather than the sclerotic lesions seen in patients.

Prostate cancer differs from many adult solid tumors in its marked dependence on the TME. This dependence has made it difficult to develop models reflective of human prostate cancer. Moreover, prostate cancer cell lines are driven by autocrine growth factors in cell lines such as DU-145, which are mostly grown in the absence of TMEs [26]. Therefore, better model systems are needed that more accurately mimic the complex interaction between prostate cancer cells and the TME [27].

Patient-derived xenografts (PDX) address the complex interaction between prostate cancer cells and the TME because they include the TME host component. The potential of these models to discover targets or establish proof of principle advanced most knowledge in human–murine coclinical studies. This approach of integrating human and mouse studies has proven more informative than using single-species studies exclusively. These integrated research strategies have led to the identification of potential new targets for the treatment of bone metastases in prostate cancer [28].

A study using the PDX model MDA PCa-118b implicated fibroblast growth factor (FGF)-9 in the pathogenesis of bone metastases in prostate cancer; PDX mice treated with antibody to FGF-9 developed smaller tumors within bone and reduced ectopic bone formation compared with control mice [29]. Another study demonstrated the role of FGF in establishing bone metastases and the potential therapeutic value of treating bone metastases in prostate cancer with the FGF inhibitors dovitinib and cabozantinib, although phase 3 studies indicated that the potential will not be met with cabozantinib, at least in the population tested [23, 28, 30, 31].

Moreover, using prostate cancer PDX, investigators recently reported that FGF receptor 1 may be a mechanism of acquired resistance to cabozantinib. Cabozantinib inhibits VEGF and c-MET, in addition to FGF, and in prostate cancer studies, was shown to activate innate immunity [32]. Studies using PDX showed that cabozantinib did not inhibit prostate cancer cells, but instead, inhibited VEGF receptor 2 and c-Met expressed by endothelial cells. In addition, direct effects of cabozantinib on osteoblasts accounted for tumor inhibition, which was seen in the PDX model and in a phase 2 trial, in which 16 of the 20 enrolled patients showed ≥ 30% reduction in bone scan lesion area (the marker for tumor response) [28]. As mentioned previously, final analysis of the phase 3 studies showed that cabozantinib improved bone scan response and progression-free survival, without significantly increasing overall survival (OS) or improving pain response [30, 31].

A major theoretical advantage of using PDX models is that they are frequently established while donors are alive, and therefore can be linked prospectively with clinical disease progression [33]. In a pilot study of patients with advanced solid tumors, PDX-guided treatment resulted in 11 partial responses among 14 patients, with an objective response rate of 88% [34].

However, in practice, PDX models, though highly informative, have proven too cumbersome to be widely applicable guides to individualized therapy. Among the other disadvantages of PDX models are their inability to account for the immune microenvironment and their slow rate of growth. These limitations have led investigators to adopt a panel of models that span *in vitro* and *in vivo* system and annotated clinical tissue that need to be subjected to integrated analyses.

Other models have been used to elucidate specific aspects of metastatic CRPC. For example, a trabecular bone model was used to examine the effects of alpha-emitting therapy, such as Ra-223 on bone marrow. Such a model comprises a sphere of tissue having a shell that is composed of bone (osteoblasts and other cells) surrounding a trabecular cavity that contains marrow cells. Hobbs and colleagues predicted that increased bone marrow toxicity should not result from the use of Ra-223 to treat bone metastases of CRPC, although they noted that the deposition of daughter nuclides in bone could not be accurately assessed by the model [35].

## Preclinical data demonstrating Ra-223 efficacy for treating bone metastases in castrate-resistant prostate cancer

Since its discovery by the Curies, radium has been known to damage tissue and, therefore, could be used for cancer therapy. An alkaline earth element, radium behaves similarly to calcium and combines with hydroxyapatite in bone. Ra-223 emits primarily alpha particles, which are particles with poor tissue penetration that can be shielded by a sheet of paper [36]. Preclinical studies in mice showed that the parenteral administration of Ra-223 resulted in its accumulation predominantly in bone tissue (femurae and skull) and preferentially at sites of increased osteoblastic activity, with some deposition in soft tissues (large and small intestines and spleen). Studies also showed that measurable daughter nuclides (e.g., Bi-211) were likewise retained in bone, although some migration to soft tissue was noted (< 2%) [37]. These results implied that the majority of the energy from Ra-223 decay could be expected to remain within bone tissue and immediately adjacent to the bone surface.

Because overexpression of growth factors, such as epidermal growth factor, confers some radioresistance to cancer lines, a series of mouse xenograft studies were performed to determine the extent of prostate cancer cell sensitivity to alpha-particle, beta-particle, or gamma-particle emitters. The findings were as expected: both androgen-sensitive and androgen-resistant prostate cancer cells responded nearly equally to irradiation by Ra-223, Lu-177 (beta-particle emitter), or Cs-137 (gamma-particle emitter), providing the rationale for using such emitters in the clinic for all disease states [38].

Other studies in tumor-bearing mice showed that the majority of the Ra-223 accumulation occurred in bone, with some deposition in kidneys, intestines, and spleen. Within bone, most of the Ra-223 deposition occurred at sites of increased osteoblastic activity. Ra-223 did not accumulate within tumor cells but rather along the apposite bone surfaces [39].

In prostate cancer xenograft models using LNCaP (androgen-sensitive) and LuCaP 58 (abiraterone-resistant) cell lines, Ra-223 showed inhibitory effects on experimental bone metastases, while preserving bone structure [14]. Intratibial injection of either cell line in mice set up tumors mimicking bone metastases. Mice were given Ra-223 based on serum PSA levels, and analyses were performed to determine the effect of treatment on several bone and tumor parameters. Ra-223 reduced osteoblastic bone growth, while preserving bone architecture and bone volume. Moreover, Ra-223 lowered serum PSA levels and reduced tumor volumes and markers of bone metabolism. Double-stranded DNA breaks were also detected in osteoblasts and osteoclasts within tumor lesions. These results indicate a dual mechanism of action of Ra-223 for inhibiting both tumor growth and pathological new bone formation adjacent to tumor foci [14].

## Direct and indirect effects of Ra-223 on bone metastases in castrate-resistant prostate cancer

Radiation has long been a mainstay treatment for bone metastases. External-beam radiation therapy (EBRT) provides pain relief, but can damage healthy tissue and tumor tissue. Bone-seeking radiopharmaceuticals, such as Ra-223 or strontium (Sr)-89, were developed to treat multiple lesions with systemic therapy, and thus complement EBRT. Both Ra and Sr are elements that displace Ca in mineralized bone. Sr-89 is a beta emitter, whereas Ra-223, as noted previously, is an alpha emitter. [40] These radiopharmaceuticals cause little myelosuppression. The beta particles emitted by Sr-89 have longer path lengths (100 mm) than the alpha particles of Ra-223 (< 100 μm), which means that the energy of Sr-89 may exert more collateral damage to bone marrow than would Ra-223 [37].

Because alpha particles are helium nuclei and thus relatively heavy charged particles, alpha emitters, such as Ra-223, deliver high-linear energy transfer radiation into cells within the short path length, resulting in nonreparable double-stranded DNA breaks. The sum of these types of damage may limit the regrowth of dormant tumor cells within bone.

Additional advantages of alpha particles include no need for elaborate shielding, and the relatively short half-life (11.4 days) of Ra-223 specifically means that waste disposal poses fewer challenges than would be the case with long-lived nuclides. Moreover, compared with beta-emitting nuclides, particles produced by alpha-emitting nuclides are more energetic and have high-linear energy transfer. Alpha particles cause predominantly nonreparable double-stranded breaks in DNA and, thus, are more toxic to tissue than beta particles [40].

**Fig. 2:**
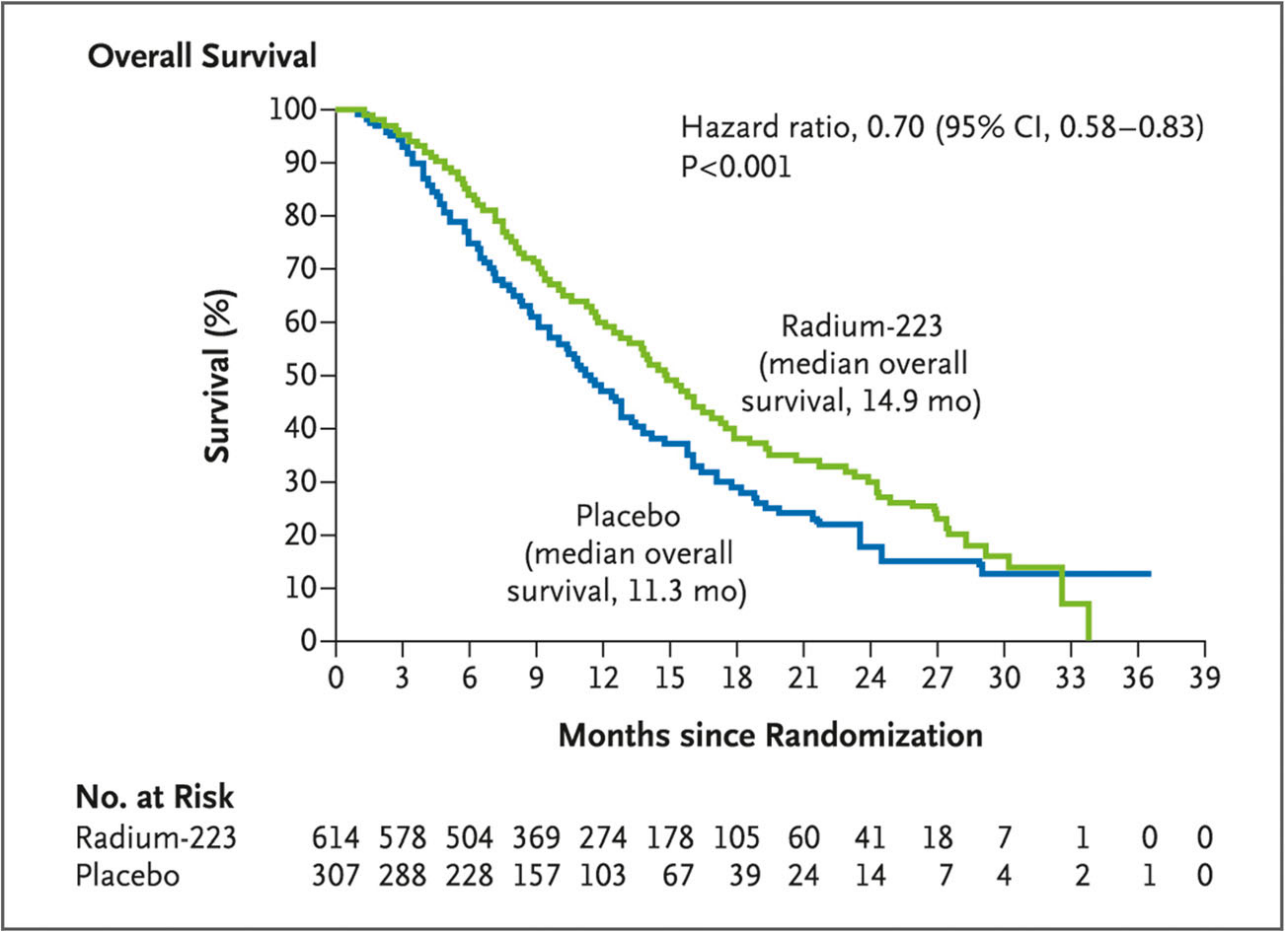
Kaplan–Meier estimates of overall survival (OS) show that treatment with Ra-223 increased the median OS significantly relative to placebo, from 11.3 to 14.9 months (*P* < .001; hazard ratio, 0.70; 95% confidence interval, 0.58–0.83). Republished with permission of [15]. Permission conveyed through Copyright Clearance Center, Inc.

Clinical data show that Ra-223 contributes to an increase in survival expectations, in addition to its effects on bone metastases in CRPC [13]. Results of the ALSYMPCA (ALpharadin in SYMPtomatic Prostate Cancer; NCT00699751) trial showed that Ra-223 improved OS in patients with CRPC, irrespective of previous treatment with docetaxel chemotherapy [15, 41]. Treatment with Ra-223 significantly increased the median OS to 14.9 months compared with 11.3 months for placebo (*P* < .001) and reduced the risk for death by 30% among patients who received standard of care (Fig. 2) [15]. When patients were stratified based on whether they previously received docetaxel, Ra-223 prolonged the median OS for both subgroups (hazard ratio [HR], 0.70 for patients who had previous docetaxel use; *P* = .002; HR, 0.69 for patients who had no previous docetaxel use, *P* = .01) [41]. In both studies, Ra-223 was well tolerated [15, 41].

Having established the efficacy of Ra-223 as an effective single agent in advanced, late-stage CRPC, current trials focus on the earlier use of Ra-223 alongside other agents, such as abiraterone (NCT02043678) and enzalutamide (NCT02199197). Data from an expanded access program that included patients with metastatic CRPC receiving a variety of treatments showed that Ra-223 could be combined with enzalutamide or abiraterone, even if patients are symptomatic, and that Ra-223 combined with antiandrogen receptor therapy modulates the bone microenvironment [42, 43]. The results of the ongoing clinical trials should reveal the effects of the combination treatment on prolonged survival.

Radium therapy likely has effects beyond direct effects on tumor cells; Ra-223 treatment may modulate immune responses similar to adjuvant therapy or modify the tumor microenvironment in such a manner as to allow more effective T cell ingress. In some studies, subtherapeutic doses of Ra-223 enhanced cell killing by T cells and expression of major histocompatibility markers on antigen-presenting cells (indirectly enhancing T cell activity) [44, 45]. Moreover, memory cells, but not naïve T cells, were found to be radioresistant, an observation that could be exploited in combining immunotherapy with Ra-223 treatment [44].

Even more important in today’s treatment landscape is the observed interaction between radiotherapy and the checkpoint inhibitor anti-programmed cell death ligand 1 (anti-PD-L1) antibody in animal models. In preclinical studies, researchers showed that PD-L1 expression was increased following exposure to ionizing radiation and that the administration of anti-PD-L1 antibody synergized with radiation treatment to reduce suppressor cells localized to the tumor environment. This, in turn, enhanced antitumor cytotoxic T cell function as a direct result of this synergy [46].

## Unanswered questions and future directions

Ra-223 has been shown to prolong survival and improve symptoms in men with metastatic CRPC who have symptomatic bone metastases. These benefits have been associated with a consistent decline in serum alkaline phosphatase levels, without a proportional decline in serum PSA levels or limiting myelosuppression. The high-linear energy transfer of alpha particles emitted by Ra-223 causes localized tissue destruction [47]. This low level of myelosuppression allows Ra-223 to be combined with chemotherapy having some level of bone marrow toxicity [47]. In addition, Ra-223 should accumulate preferentially where there is osteoblastic growth surrounding osteolytic lesions and therefore deliver its therapeutic effects in targeted anatomical regions [47].

Perhaps, the most promising is the potential for combinations of bone-targeted therapies to prevent progression as opposed to solely treating existing metastases. Exploring rational combinations of approved agents (i.e., second-generation antiandrogen drugs) or agents in development is a high-priority area of study that builds on the progress made to date. One trial, ERA-223, explores the value of adding Ra-223 to abiraterone (plus pedisone or prednisolone; NCT02043678). Patient accrual has completed (806 enrolled), and results are expected in the next 2 years.

One future direction with Ra-223 therapy may be to combine it with immunotherapy. One trial that is underway (NCT03093428) will determine whether the combination of Ra-223 and pembrolizumab (US Food and Drug Administration [FDA]-approved anti-PD-L1 antibody) has shown early promise in patients with metastatic CRPC. In this trial, patients who previously received enzalutamide or abiraterone will receive Ra-223 plus pembrolizumab either concurrently or on a staggered regimen. The primary end point is the extent of immune-cell (e.g., CD8+ and CD4+ T cells) infiltration in bone biopsy specimens from baseline to 8 weeks following the start of study treatment. The secondary end points include safety, tolerability, and tumor efficacy metrics. Another clinical trial (NCT02814669) will evaluate the safety and tolerability of atezolizumab plus Ra-223 in metastatic CRPC following treatment with an androgen pathway inhibitor. Atezolizumab, like pembrolizumab, is an FDA-approved anti-PD-L1 antibody.

As factors involved in bone metastases are identified and novel agents targeted to these factors are recognized, new trials are anticipated to test combinations of new agents with Ra-223 in CRPC. One pathway that could yield new agents is the TBX2-WNT signaling axis. TBX2 is a transcription factor that is overexpressed in bone metastases of CRPC, which acts through the WNT signaling pathway shown to be involved in prostate cancer progression and bone metastases. Blocking TBX2 reduced bone metastases and tumor growth in mouse prostate cancer xenograft models [48].

## Conclusion

Bone metastases in CRPC arise from complex interactions among factors that drive prostate cancer progression and lead to more bone metastases. Among the treatments for bone metastases, Ra-223 has unique properties that enhance not only its capability to inhibit bone metastases, but also its ability to influence the bone microenvironment in ways that may augment antitumor activities within the body and thereby prolong OS. Results of ongoing and future trials will help determine optimal treatment combinations and sequencing of effective therapies to prevent and treat bone metastases in CRPC.

## References

[1] National Cancer Institute. (2017). SEER Cancer Stat Facts: prostate cancer. http://seer.cancer.gov/statfacts/html/prost.html. Accessed August 9, 2017.

[2] Sweeney CJ, Chen Y-H, Carducci M, Liu G, Jarrard DF, Eisenberger M (2015). Chemohormonal therapy in metastatic hormone-sensitive prostate cancer. New England Journal of Medicine.

[3] Aparicio AM, Shen L, Tapia ELN, Lu J-F, Chen H-C, Zhang J (2016). Combined tumor suppressor defects characterize clinically defined aggressive variant prostate cancers. Clinical Cancer Research.

[4] Tzelepi V, Zhang J, Lu J-F, Kleb B, Wu G, Wan X (2012). Modeling a lethal prostate cancer variant with small-cell carcinoma features. Clinical Cancer Research.

[5] Mu P, Zhang Z, Benelli M, Karthaus WR, Hoover E, Chen C-C (2017). SOX2 promotes lineage plasticity and antiandrogen resistance in TP53- and RB1-deficient prostate cancer. Science (New York, N.Y.).

[6] Grasso CS, Wu Y-M, Robinson DR, Cao X, Dhanasekaran SM, Khan AP (2012). The mutational landscape of lethal castration-resistant prostate cancer. Nature.

[7] Logothetis CJ, Gallick GE, Maity SN, Kim J, Aparicio A, Efstathiou E (2013). Molecular classification of prostate cancer progression: foundation for marker-driven treatment of prostate cancer. Cancer Discovery.

[8] Ottewell PD, Wang N, Meek J, Fowles CA, Croucher PI, Eaton CL (2014). Castration-induced bone loss triggers growth of disseminated prostate cancer cells in bone. Endocrine-Related Cancer.

[9] Kimura Y, Matsugaki A, Sekita A, Nakano T (2017). Alteration of osteoblast arrangement via direct attack by cancer cells: new insights into bone metastasis. Scientific Reports.

[10] Kingsley LA, Fournier PGJ, Chirgwin JM, Guise TA (2007). Molecular biology of bone metastasis. Molecular Cancer Therapeutics.

[11] Eiro N, Fernandez-Gomez J, Sacristán R, Fernandez-Garcia B, Lobo B, Gonzalez-Suarez J (2017). Stromal factors involved in human prostate cancer development, progression and castration resistance. Journal of Cancer Research and Clinical Oncology.

[12] Navone NM, Rodriguez-Vargas MDC, Benedict WF, Troncoso P, McDonnell TJ, Zhou J-H (2000). TabBO: a model reflecting common molecular features of androgen-independent prostate cancer. Clinical Cancer Research.

[13] Liepe K, Shinto A (2016). From palliative therapy to prolongation of survival: (223)RaCl(2) in the treatment of bone metastases. Therapeutic Advances in Medical Oncology.

[14] Suominen, M. I., Fagerlund, K. M., Rissanen, J. P., Konkol, Y. M., Morko, J. P., Peng, Z., et al. (2017). Radium-223 inhibits osseous prostate cancer growth by dual targeting of cancer cells and bone microenvironment in mouse models. *Clinical Cancer Research*, In press. 10.1158/1078-0432.ccr-16-2955. 10.1158/1078-0432.CCR-16-2955 PMC5540794 28364014

[15] Parker C, Nilsson S, Heinrich D, Helle SI, O'Sullivan JM, Fosså SD (2013). Alpha emitter radium-223 and survival in metastatic prostate cancer. New England Journal of Medicine.

[16] Wang N, Docherty FE, Brown HK, Reeves KJ, Fowles ACM, Ottewell PD (2014). Prostate cancer cells preferentially home to osteoblast-rich areas in the early stages of bone metastasis: evidence from in vivo models. Journal of Bone and Mineral Research.

[17] Luo Y, He D-L, Ning L, Shen S-L, Li L, Li X (2006). Over-expression of hypoxia-inducible factor-1α increases the invasive potency of LNCaP cells in vitro. BJU International.

[18] Brahme A (2011). Accurate description of the cell survival and biological effect at low and high doses and LET’s. Journal of Radiation Research.

[19] Fidler IJ (2003). The pathogenesis of cancer metastasis: the ‘seed and soil’ hypothesis revisited. Nature Reviews. Cancer.

[20] Jin J-K, Dayyani F, Gallick GE (2011). Steps in prostate cancer progression that lead to bone metastasis. International Journal of Cancer.

[21] Dayyani F, Gallick GE, Logothetis CJ, Corn PG (2011). Novel therapies for metastatic castrate-resistant prostate cancer. JNCI Journal of the National Cancer Institute.

[22] Fournier PGJ, Juárez P, Jiang G, Clines GA, Niewolna M, Kim HS (2015). The TGF-b2; signaling regulator PMEPA1 suppresses prostate cancer metastases to bone. Cancer Cell.

[23] Wan X, Corn PG, Yang J, Palanisamy N, Starbuck MW, Efstathiou E (2014). Prostate cancer cell–stromal cell crosstalk via FGFR1 mediates antitumor activity of dovitinib in bone metastases. Science Translational Medicine.

[24] Logothetis CJ, Lin S-H (2005). Osteoblasts in prostate cancer metastasis to bone. Nature Reviews. Cancer.

[25] Fizazi K, Yang J, Peleg S, Sikes CR, Kreimann EL, Daliani D (2003). Prostate cancer cells-osteoblast interaction shifts expression of growth/survival-related genes in prostate cancer and reduces expression of osteoprotegerin in osteoblasts. Clinical Cancer Research.

[26] Connolly JM, Rose DP (1991). Autocrine regulation of DU145 human prostate cancer cell growth by epidermal growth factor-related polypeptides. The Prostate.

[27] Rea D, del Vecchio V, Palma G, Barbieri A, Falco M, Luciano A (2016). Mouse models in prostate cancer translational research: from xenograft to PDX. BioMed Research International.

[28] Varkaris A, Corn PG, Parikh NU, Efstathiou E, Song JH, Lee Y-C (2016). Integrating murine and clinical trials with cabozantinib to understand roles of MET and VEGFR2 as targets for growth inhibition of prostate cancer. Clinical Cancer Research.

[29] Li ZG, Mathew P, Yang J, Starbuck MW, Zurita AJ, Liu J (2008). Androgen receptor–negative human prostate cancer cells induce osteogenesis in mice through FGF9-mediated mechanisms. The Journal of Clinical Investigation.

[30] Smith, M., de Bono, J. S., Sternberg, C. S., Le Moulec, S., Oudard, S., De Giorgi, U., et al. (2015). Final analysis of COMET-1: cabozantinib (cabo) versus prednisone (pred) in metastatic castration-resistant prostate cancer (mCRPC) patients (pts) previously treated with docetaxel (D) and abiraterone (A) and/or enzalutamide (E). *J Clin Oncol, 33*(Suppl 7), Abstract 139.

[31] Basch, E. M., Scholz, M. C., De Bono, J. S., Vogelzang, N. J., De Souza, P. L., Marx, G. M., et al. (2015). Final analysis of COMET-2: cabozantinib (Cabo) versus mitoxantrone/prednisone (MP) in metastatic castration-resistant prostate cancer (mCRPC) patients (pts) with moderate to severe pain who were previously treated with docetaxel (D) and abiraterone (A) and/or enzalutamide (E). *J Clin Oncol, 33*(Suppl 7), Abstract 141.

[32] Sidaway P (2017). Prostate cancer: cabozantinib activates innate immunity. [research highlight]. Nature Reviews. Urology.

[33] Hidalgo M, Amant F, Biankin AV, Budinská E, Byrne AT, Caldas C (2014). Patient derived xenograft models: an emerging platform for translational cancer research. Cancer Discovery.

[34] Hidalgo M, Bruckheimer E, Rajeshkumar NV, Garrido-Laguna I, De Oliveira E, Rubio-Viqueira B (2011). A pilot clinical study of treatment guided by personalized tumorgrafts in patients with advanced cancer. Molecular Cancer Therapeutics.

[35] Hobbs RF, Song H, Watchman CJ, Bolch WE, Aksnes A-K, Ramdahl T (2012). A bone marrow toxicity model for ^223^Ra alpha-emitter radiopharmaceutical therapy. Physics in Medicine and Biology.

[36] Vapiwala N, Glatstein E (2013). Fighting prostate cancer with radium-223—not your Madame’s isotope. New England Journal of Medicine.

[37] Henriksen G, Fisher DR, Roeske JC, Bruland ØS, Larsen RH (2003). Targeting of osseous sites with α-emitting 223Ra: comparison with the β-emitter 89Sr in mice. Journal of Nuclear Medicine.

[38] Elgqvist J, Timmermand OV, Larsson E, Strand S-E (2016). Radiosensitivity of prostate cancer cell lines for irradiation from beta particle-emitting radionuclide 177Lu compared to alpha particles and gamma rays. Anticancer Research.

[39] Abou DS, Ulmert D, Doucet M, Hobbs RF, Riddle RC, Thorek DLJ (2016). Whole-body and microenvironmental localization of radium-223 in naïve and mouse models of prostate cancer metastasis. JNCI: Journal of the National Cancer Institute.

[40] Bruland ØS, Nilsson S, Fisher DR, Larsen RH (2006). High-linear energy transfer irradiation targeted to skeletal metastases by the α-emitter ^223^Ra: adjuvant or alternative to conventional modalities?. Clinical Cancer Research.

[41] Hoskin P, Sartor O, O'Sullivan JM, Johannessen DC, Helle SI, Logue J (2014). Efficacy and safety of radium-223 dichloride in patients with castration-resistant prostate cancer and symptomatic bone metastases, with or without previous docetaxel use: a prespecified subgroup analysis from the randomised, double-blind, phase 3 ALSYMPCA trial. The Lancet Oncology.

[42] Saad F, Carles J, Gillessen S, Heidenreich A, Heinrich D, Gratt J (2016). Radium-223 and concomitant therapies in patients with metastatic castration-resistant prostate cancer: an international, early access, open-label, single-arm phase 3b trial. The Lancet Oncology.

[43] Yeku O, Slovin SF (2016). Radium-223 and concomitant therapies: prospects and prudence. Translational Andrology and Urology.

[44] Miyahira AK, Morris M, Soule HR, Group, P. C. F. R.-S. W (2017). Meeting report from the Prostate Cancer Foundation Scientific Working Group on radium-223. The Prostate.

[45] Gelbard A, Garnett CT, Abrams SI, Patel V, Gutkind JS, Palena C (2006). Combination chemotherapy and radiation of human squamous cell carcinoma of the head and neck augments CTL-mediated lysis. Clinical Cancer Research : an official journal of the American Association for Cancer Research.

[46] Deng L, Liang H, Burnette B, Beckett M, Darga T, Weichselbaum RR (2014). Irradiation and anti–PD-L1 treatment synergistically promote antitumor immunity in mice. The Journal of Clinical Investigation.

[47] Coleman R (2016). Treatment of metastatic bone disease and the emerging role of radium-223. Seminars in Nuclear Medicine.

[48] Nandana S, Tripathi M, Duan P, Chu C-Y, Mishra R, Liu C (2017). Bone metastasis of prostate cancer can be therapeutically targeted at the TBX2–WNT signaling axis. Cancer Research.

